# Wogonin Ameliorates the Oxidative Stress, Apoptosis, and Extracellular Matrix Degradation of Nucleus Pulposus Cells Mediated by *Cutibacterium acnes* via the MAPK Signaling Pathway: An In Vivo and In Vitro Study

**DOI:** 10.3390/ijms27104249

**Published:** 2026-05-10

**Authors:** Jingwen Jia, Yuxuan Bai, Mingtao Zhang, Shuanhu Lei, Mingdong Ma, Kangyong Gao, Xuewen Kang

**Affiliations:** Department of Orthopaedics, Lanzhou University Second Hospital, Lanzhou 730030, China; 220220904251@lzu.edu.cn (J.J.);

**Keywords:** *Cutibacterium acnes*, low back pain, intervertebral disc degeneration, nucleus pulposus, oxidative stress, Wogonin, in vivo and in vitro study

## Abstract

Intervertebral disc degeneration (IDD) is a fundamental pathological basis of low back pain, yet its pathogenic mechanisms remain incompletely understood. Infection by low-virulence anaerobic bacteria has recently been recognized as a potential etiological factor. In this study, *Cutibacterium acnes* (*C. acnes*) was detected in 13.7% of degenerated intervertebral disc (IVD) tissues, and its presence was significantly associated with younger patients and Modic changes. In vitro experiments demonstrated that *C. acnes* supernatant induces oxidative stress, apoptosis, and extracellular matrix (ECM) degradation in nucleus pulposus (NP) cells in a dose-dependent manner. RNA sequencing and functional validation further indicated that these pathological effects are mediated through activation of the p38 MAPK signaling pathway. Pharmacological inhibition of p38 with the specific inhibitor BIRB-796 effectively reversed the observed cellular damage. Wogonin exhibited negligible cytotoxicity toward NP cells and significantly attenuated *C. acnes* supernatant-induced oxidative stress, apoptosis, and ECM metabolic imbalance by inhibiting the phosphorylation of p38, JNK, and ERK1/2 within the MAPK pathway. Furthermore, in vivo experiments confirmed that Wogonin alleviated disc height loss, reduced T2-weighted signal attenuation, and mitigated histological damage induced by *C. acnes* in rat models, thereby restoring the balance between ECM synthesis and degradation. Collectively, this study demonstrates for the first time that *C. acnes* supernatant exacerbates IDD through activation of the p38 MAPK signaling pathway. It further shows that Wogonin can specifically inhibit this pathway and effectively ameliorate *C. acnes*-mediated IDD damage in both in vitro and in vivo models. These findings expand the theoretical framework of infection-related mechanisms underlying IDD and identify potential therapeutic targets and candidate agents for the treatment of IDD associated with *C. acnes* infection. Low back pain is a common health issue affecting populations worldwide, with intervertebral disc degeneration as its core etiology. However, the pathogenic causes in some patients, especially young individuals, remain incompletely understood. This study found that *Cutibacterium acnes*, a low-virulence bacterium commonly colonizing human skin and mucous membranes, produces metabolic products that can induce damage to the core cells of the intervertebral disc, exacerbate disc degeneration, and this process is associated with the abnormal activation of specific cellular signaling pathways. Through clinical sample detection, cell experiments, and animal model validation, we confirmed that infection with this bacterium is closely related to young patients and specific spinal imaging changes. Meanwhile, we identified Wogonin, a natural compound extracted from Scutellaria baicalensis, which can effectively inhibit the aforementioned abnormal signaling pathways, alleviate cell damage caused by bacterial metabolic products, and improve the pathological state of intervertebral disc degeneration. This study not only reveals the role of low-virulence bacterial infection in intervertebral disc degeneration and provides a new explanation for the pathogenic mechanism in young patients but also offers a natural antibiotic-free candidate for addressing bacterial resistance. It holds significant reference value for the clinical diagnosis and treatment of spinal diseases as well as the development of related drugs.

## 1. Introduction

Low back pain (LBP) is a common clinical symptom associated with spinal disorders and is among the leading causes of reduced work capacity and impaired quality of life [1,2]. Epidemiological studies indicate that LBP occurs in high-, middle-, and low-income countries and affects individuals across all age groups, from children to older adults. This widespread prevalence has led to a substantial increase in the demand for rehabilitation services across 134 countries and regions [3,4]. Intervertebral disc degeneration (IDD) is a major contributor to spinal disorders, including intervertebral disc (IVD) herniation, spinal stenosis, and spondylolisthesis. As the disease progresses, it can lead to both acute and chronic LBP, making it a key pathological basis of LBP [5].

The normal IVD is composed of superior and inferior cartilaginous endplates, an annulus fibrosus (AF), and a central gelatinous nucleus pulposus (NP). The NP plays a crucial role in absorbing mechanical stress and maintaining the mobility of spinal segments [6]. During the progression of IDD, the NP exhibits decreased water content and degradation of the extracellular matrix (ECM), accompanied by structural damage to the AF and a reduction in IVD height. These changes ultimately lead to structural deterioration and functional impairment of the IVD [7]. Multiple factors have been implicated in the development of IDD, including oxidative stress, inflammatory responses, cellular senescence, abnormal mechanical loading, metabolic disorders, and genetic predisposition. However, the pathophysiology of IDD is highly complex and involves interactions among multiple biological and environmental factors. Consequently, its precise etiology and molecular mechanisms remain incompletely understood. A deeper understanding of the mechanisms underlying IDD is therefore essential for the development of effective therapeutic strategies [8,9,10,11].

In recent years, infections caused by low-virulence, non-pyogenic anaerobic bacteria have been proposed as potential pathogenic factors in IDD. In 2001, Stirling et al. first reported the isolation and culture of low-virulence anaerobic bacteria, particularly *Cutibacterium acnes* (*C. acnes*), from degenerated IVD tissue in *The Lancet*, drawing significant attention from the scientific community [12]. *C. acnes* is a Gram-positive facultative anaerobic bacterium that commonly colonizes human skin and mucous membranes and generally exhibits low virulence and invasiveness [13]. Previous studies have associated *C. acnes* with conditions such as bone hyperplasia, synovitis, urinary system disorders, and infections related to surgical implants [14,15,16]. Although some researchers question whether the presence of low-virulence bacteria in degenerated IVDs represents true infection or contamination, increasing evidence from bacterial culture, molecular biology, and proteomic analyses has confirmed the presence of low-virulence bacterial infections—primarily involving *C. acnes* and coagulase-negative staphylococci (CNS)—within degenerated IVD tissue [17,18,19,20,21]. Mechanistic studies further suggest that *C. acnes* may accelerate the progression of IDD by inducing pathological processes in NP cells, including oxidative stress, inflammatory responses, apoptosis, and necroptosis [22].

Redox homeostasis is essential for maintaining normal cellular function. Disruption of this balance results in oxidative stress, characterized by excessive production of reactive oxygen species (ROS) and a relative decline in antioxidant defenses, which can impair cellular integrity and function [23,24]. Recent studies have demonstrated that oxidative stress contributes to the progression of IDD through multiple molecular pathways [25,26,27]. Wang et al. reported that free fatty acids, the key pathogenic factor secreted by *C. acnes*, induce pro-inflammatory effects and oxidative stress in 3T3-L1 adipocytes [28]. However, the mechanisms by which *C. acnes* culture supernatant may promote IDD progression by inducing oxidative stress in NP cells remain unclear.

In addition, the global rate of antibiotic resistance in *C. acnes* has increased substantially, with more than 50% of strains demonstrating resistance, largely due to the inappropriate or excessive use of antibiotics [29]. Therefore, compared with conventional antibiotic therapy, the development of alternative treatment strategies that reduce antibiotic dependence or overcome drug resistance is of considerable clinical significance. Wogonin (IUPAC name: 5,7-Dihydroxy-8-methoxyflavone), a natural flavonoid compound extracted from the root of *Scutellaria baicalensis* (Huangqin), exhibits diverse pharmacological activities, including antioxidant, anti-inflammatory, anti-apoptotic, and cell cycle-regulatory effects in various diseases [30]. However, whether Wogonin can inhibit oxidative stress induced by *C. acnes*-conditioned medium in NP cells has not yet been investigated. Accordingly, the present study aims to elucidate the molecular mechanisms by which *C. acnes*-conditioned medium exacerbates oxidative stress in NP cells and contributes to IDD progression. In addition, this study explores the potential protective role of Wogonin, with the goal of providing new insights for the clinical diagnosis and treatment of *C. acnes*-associated IDD.

## 2. Results

### 2.1. Basic Characteristics of the Study Population and Bacterial Culture Results in IVD Tissues

**Figure 1:**
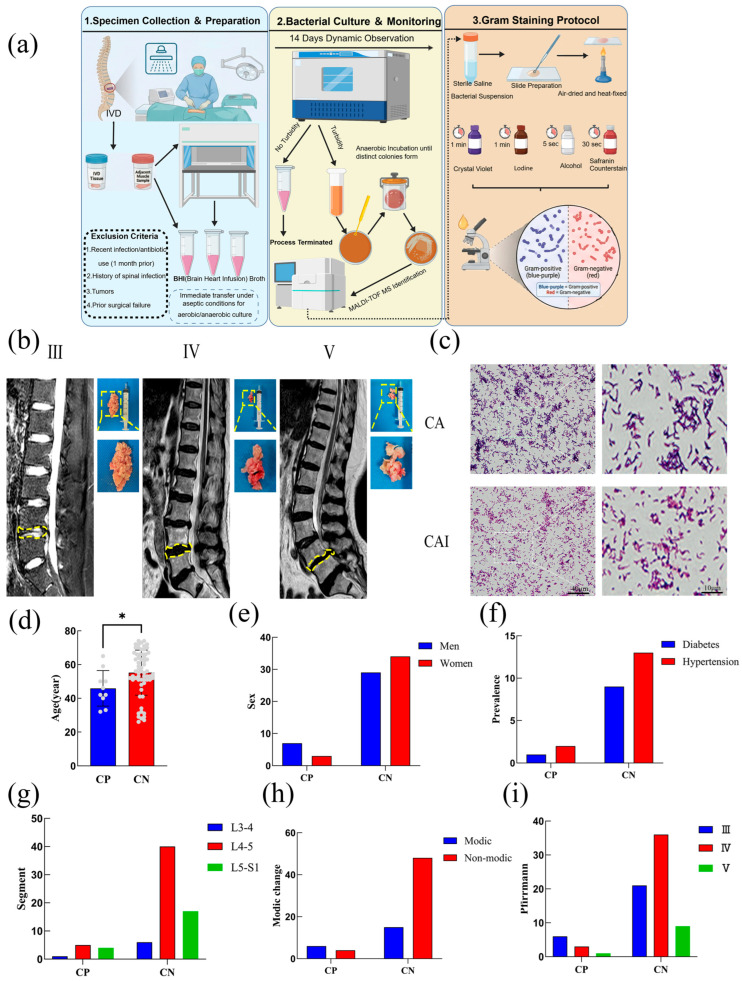
Bacterial culture and identification from intervertebral disc (IVD) tissues and correlation analysis of *Cutibacterium acnes*–positive patient characteristics. (**a**). Collection and culture procedures for IVD specimens. (**b**). Representative appearance of IVD tissues and corresponding MRI findings across different Pfirrmann grades. (**c**). Gram staining results (CA: standard *C. acnes*; CAI: tissue-derived *C. acnes*). (**d**). Correlation between *C. acnes* positivity and patient age. (**e**). Correlation between *C. acnes* positivity and sex. (**f**). Correlation between *C. acnes* positivity and the presence of diabetes and hypertension. (**g**). Correlation between *C. acnes* positivity and affected segments of intervertebral disc degeneration (IDD). (**h**). Correlation between *C. acnes* positivity and Modic changes. (**i**). Correlation between *C. acnes* positivity and Pfirrmann grade (CP: *C. acnes*–positive; CN: *C. acnes*–negative). * *p* < 0.05.

Based on the predefined inclusion and exclusion criteria, a total of 73 patients diagnosed with IDD were enrolled in this study (Table 1). Aerobic and anaerobic cultures were performed on IVD tissues from all patients (Figure 1a,b). Bacteria were detected in 10 cases, corresponding to a positivity rate of 13.7%. Gram staining showed that the morphology of *C. acnes* isolated from IVD tissues was consistent with that of standard strains observed under oil immersion microscopy (Figure 1c). Correlation analysis demonstrated a significant association between *C. acnes* positivity and patient age, with a higher positivity rate observed in younger patients (*p* < 0.05). A significant correlation was also identified with Modic changes, as *C. acnes* positivity was more frequent among patients presenting these changes (*p* < 0.05). In contrast, no significant associations were observed between *C. acnes* positivity and gender, diabetes, hypertension, degenerated segments, or Pfirrmann grade (Table 1; Figure 1d–i).

### 2.2. Effects of C. acnes Supernatant on the Activity, Oxidative Stress, Apoptosis, and ECM Degradation of Rat NP Cells

#### 2.2.1. Effects of Different Concentrations of *C. acnes* Supernatant on NP Activity

**Figure 2:**
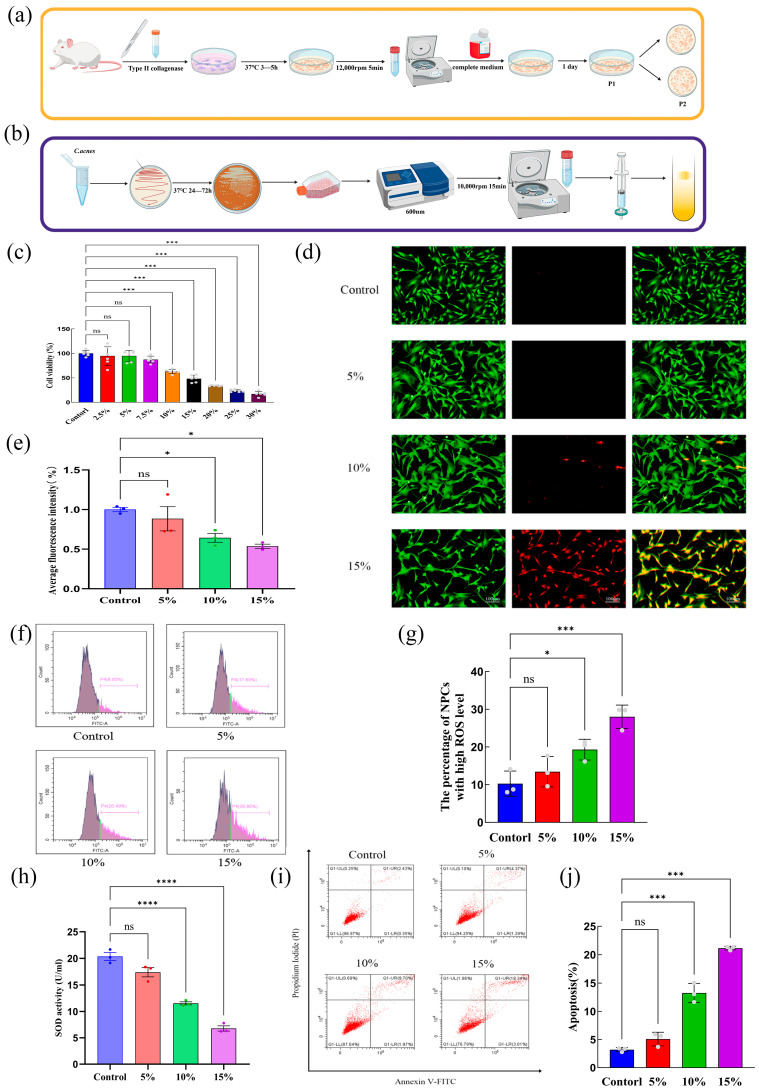
Effects of different concentrations of *C. acnes* supernatant on viability, oxidative stress, and apoptosis in rat nucleus pulposus (NP) cells. (**a**). Schematic diagram of primary rat NP cell isolation. (**b**). Preparation of *C. acnes* culture supernatant. (**c**). Effects of different concentrations of *C. acnes* supernatant on NP cell viability. (**d**). Dead-cell staining of NP cells after exposure to *C. acnes* supernatant. (**e**). Quantitative analysis of dead-cell staining. (**f**). Flow cytometric analysis of reactive oxygen species (ROS) levels following treatment with *C. acnes* supernatant. (**g**). Quantification of ROS levels. (**h**). Superoxide dismutase (SOD) activity following *C. acnes* supernatant treatment. (**i**). Flow cytometric analysis of apoptosis. (**j**). Quantitative analysis of apoptosis. ns: No statistical significance, * *p* < 0.05, *** *p* < 0.001, **** *p* < 0.0001.

Primary NP cells were successfully isolated from rats, and different concentrations of *C. acnes* supernatant were prepared (Figure 2a,b). The CCK-8 assay showed that treatment with 10% concentration of *C. acnes* supernatant significantly inhibited NP cell activity, and cell activity declined further as the concentration increased (*p* < 0.05) (Figure 2c). Live/dead cell staining produced consistent results (Figure 2d,e), confirming that higher concentrations of *C. acnes* supernatant exert significant cytotoxic effects on NP cells.

#### 2.2.2. Effects of Different Concentrations of *C. acnes* Supernatant on Oxidative Stress, Apoptosis, and ECM Degradation in NP Cells

Flow cytometry and SOD activity assays demonstrated that treatment with 10% *C. acnes* supernatant significantly increased intracellular ROS levels while decreasing SOD activity. With increasing concentrations of the supernatant, ROS levels continued to rise, and SOD activity further declined (*p* < 0.05) (Figure 2f–h). These findings indicate that *C. acnes* supernatant induces oxidative stress in NP cells beginning at a concentration of 10%, primarily by enhancing ROS production and suppressing antioxidant enzyme activity.

**Figure 3:**
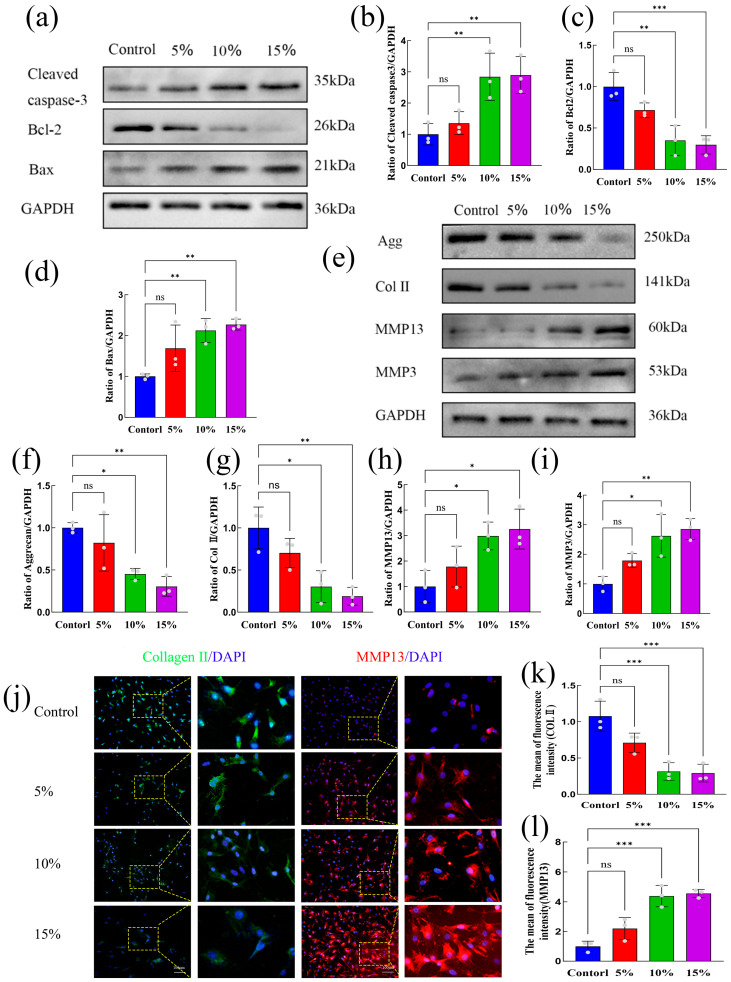
Effects of different concentrations of *C. acnes* supernatant on apoptosis and extracellular matrix (ECM) degradation in rat NP cells. (**a**). Western blot analysis of apoptosis-related proteins (cleaved caspase-3, Bax, and Bcl-2). (**b**–**d**). Quantitative analysis of apoptosis-related protein expression. (**e**). Western blot analysis of ECM synthesis and degradation markers (aggrecan, collagen II, MMP-13, and MMP-3). (**f**–**i**). Quantitative analysis of ECM-related protein expression. (**j**–**l**). Immunofluorescence detection and quantitative analysis of ECM-related markers. ns: No statistical significance, * *p* < 0.05, ** *p* < 0.01, *** *p* < 0.001.

Apoptosis analysis by flow cytometry showed that the proportion of apoptotic cells increased significantly with higher concentrations of *C. acnes* supernatant, with statistically significant differences observed at 10% (*p* < 0.05) (Figure 2i,j). Western blot analysis further revealed that *C. acnes* supernatant upregulated the expression of the pro-apoptotic proteins cleaved caspase-3 and Bax while downregulating the anti-apoptotic protein Bcl-2. These changes were statistically significant at a concentration of 10% (*p* < 0.05) (Figure 3a–d), confirming that *C. acnes* supernatant promotes apoptosis in NP cells.

Analyses of ECM metabolism showed that exposure to *C. acnes* supernatant significantly decreased the expression of ECM synthesis-related proteins, including aggrecan and collagen II, while increasing the expression of matrix degradation enzymes MMP-13 and MMP-3. These changes became statistically significant at 10% (*p* < 0.05) and were more pronounced at higher concentrations (Figure 3e–i). Immunofluorescence analysis further supported the Western blot results, demonstrating reduced collagen II expression and increased MMP-13 expression (Figure 3j–l). Based on these findings, a concentration of 10% *C. acnes* supernatant was selected for use in subsequent experiments.

### 2.3. Study on the Mechanism of C. acnes Supernatant-Mediated Damage to NP Cells

#### 2.3.1. Activation of the MAPK Signaling Pathway Following *C. acnes* Supernatant Treatment

**Figure 4:**
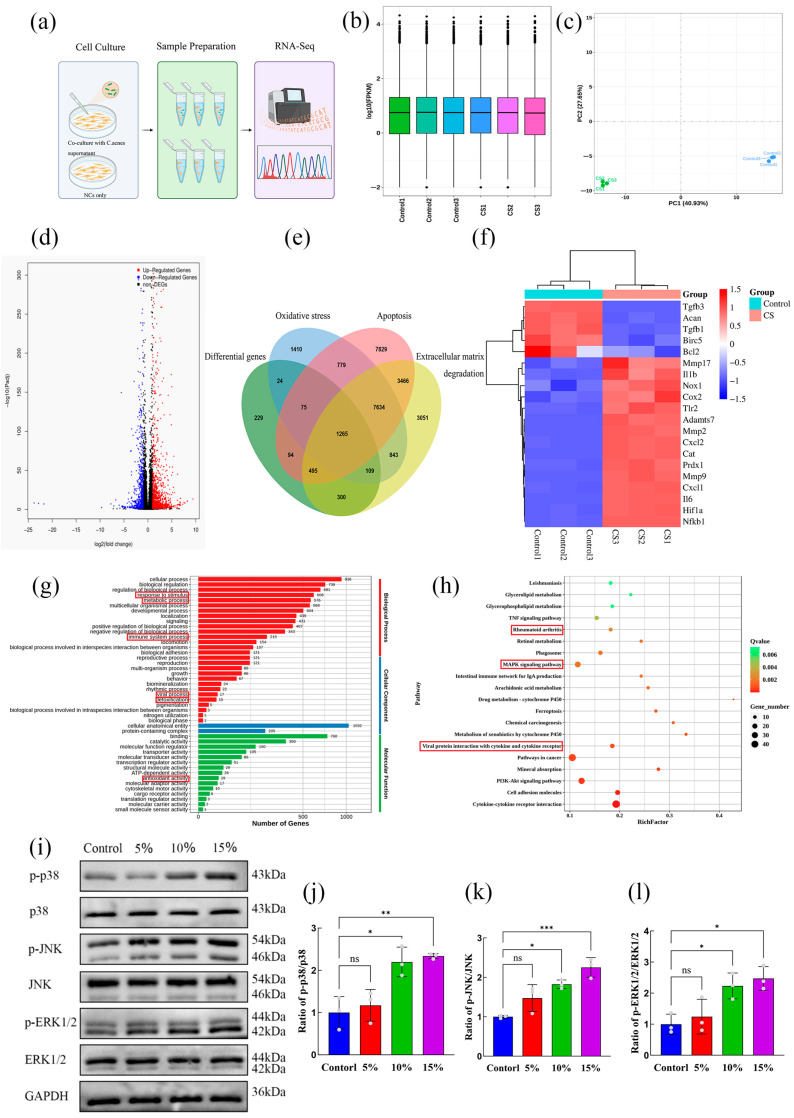
Transcriptomic analysis of rat NP cells following treatment with different concentrations of *C. acnes* supernatant. (**a**). Schematic overview of the transcriptomic sequencing workflow. (**b**). Box plot of FPKM expression levels. (**c**). Principal component analysis (PCA). (**d**). Volcano plot of differentially expressed genes. (**e**). Venn diagram of gene overlap among groups. (**f**). Heatmap of differentially expressed genes. (**g**). Gene Ontology (GO) enrichment distribution of differentially expressed genes. (**h**). KEGG pathway enrichment analysis of differentially expressed genes. (**i**–**l**). Western blot analysis and quantification of MAPK signaling pathway proteins (p-p38, p38, p-JNK, JNK, p-ERK1/2, and ERK1/2). ns: No statistical significance, * *p* < 0.05, ** *p* < 0.01, *** *p* < 0.001.

To investigate the molecular mechanisms underlying *C. acnes* supernatant-induced damage in NP cells, RNA sequencing was performed on cells treated with 10% *C. acnes* supernatant, compared to untreated controls (Figure 4a). Box plot analysis of FPKM expression levels indicated high-quality RNA samples (Figure 4b). Principal component analysis (PCA) demonstrated clear separation between treated and control groups, with minimal intra-group variation (Figure 4c). Volcano plot analysis identified 2143 differentially expressed genes (DEGs), including 1346 upregulated and 797 downregulated genes (Figure 4d). Venn diagram analysis revealed that 1265 DEGs were enriched in pathways associated with oxidative stress, apoptosis, and ECM degradation (Figure 4e). Hierarchical clustering of DEGs indicated enrichment in inflammation-related genes (e.g., *IL-1β*, *IL-6*, *NFKB1*), oxidative stress-related genes (e.g., *NOX1*, *HIF1α*), apoptosis-related genes (e.g., *BCL2*, *BIRC5*), ECM degradation-related genes (e.g., *Mmp2*, *Mmp9*), and chemokines (e.g., *Cxcl1*, *Cxcl2*) (Figure 4f). Gene Ontology (GO) analysis suggested that *C. acnes* supernatant predominantly activates immune and inflammatory responses, as well as responses to bacterial secretions (Figure 4g). KEGG pathway analysis revealed significant enrichment of DEGs in inflammatory signaling pathways, particularly MAPK and TNF pathways (Figure 4h). Western blot validation confirmed that phosphorylated proteins in the MAPK pathway—p-p38, p-JNK, and p-ERK—were upregulated in a dose-dependent manner following *C. acnes* supernatant treatment, with statistically significant changes observed at 10% concentration (*p* < 0.05) (Figure 4i–l). These findings indicate that *C. acnes* supernatant may mediate NP cell damage via activation of the MAPK signaling pathway.

#### 2.3.2. Effects of *C. acnes* Supernatant on Oxidative Stress, Apoptosis, and ECM Degradation in Rat NP Cells Through the MAPK Signaling Pathway

**Figure 5:**
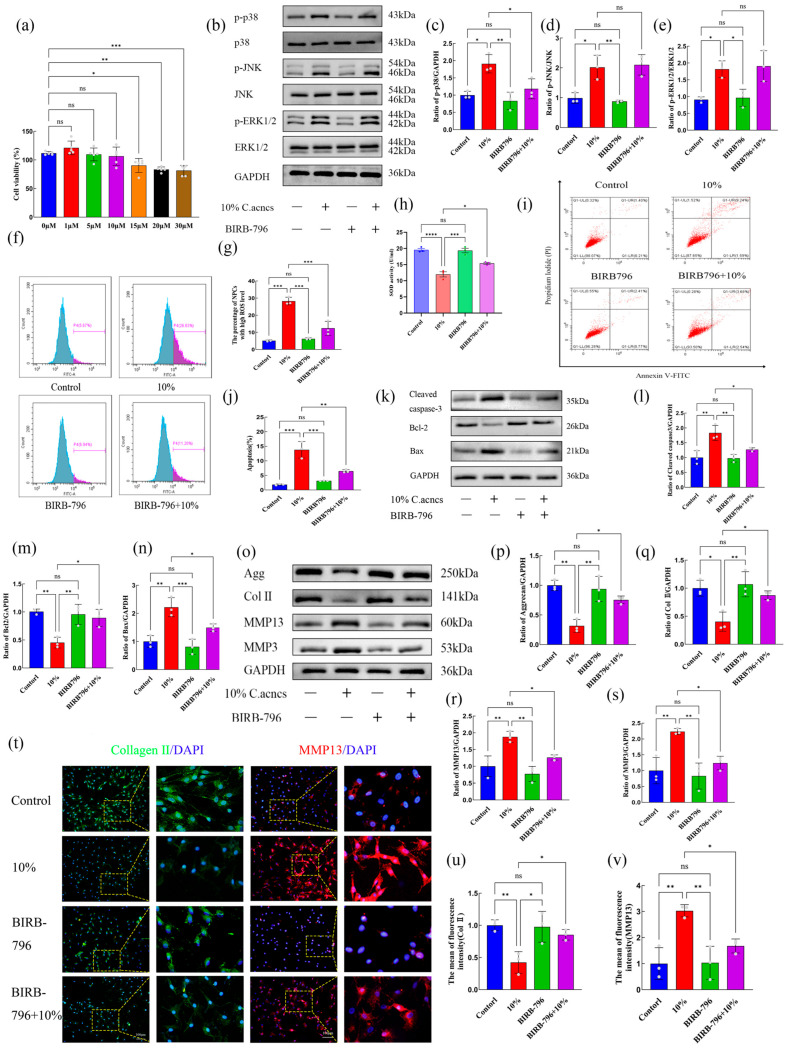
Effects of inhibiting the p38 MAPK signaling pathway on *C. acnes* supernatant-induced damage in rat NP cells. (**a**). Effect of the p38 MAPK inhibitor BIRB-796 on NP cell viability. (**b**–**e**). Western blot analysis and quantification of MAPK pathway-related proteins after BIRB-796 treatment. (**f**–**h**). ROS levels, quantitative analysis, and SOD activity following BIRB-796 treatment. (**i**–**n**). Flow cytometric analysis and quantification of apoptosis, and Western blot analysis of apoptosis-related proteins after BIRB-796 treatment. (**o**–**v**). Western blot analysis, quantitative analysis, and immunofluorescence detection of ECM-related markers following BIRB-796 treatment. ns: No statistical significance, * *p* < 0.05, ** *p* < 0.01, *** *p* < 0.001, **** *p* < 0.0001.

To determine the functional contribution of the MAPK pathway, NP cells were treated with the p38 MAPK-specific inhibitor BIRB-796. CCK-8 assays demonstrated that BIRB-796 at concentrations of 0–50 μM had no significant effect on cell viability, whereas concentrations above 75 μM significantly reduced viability (*p* < 0.05) (Figure 5a). Accordingly, 10 μM BIRB-796 was used for subsequent experiments. Western blot analysis showed that BIRB-796 significantly inhibited the *C. acnes* supernatant-induced increase in p-p38 phosphorylation (*p* < 0.05) but had no significant effect on p-JNK or p-ERK1/2 phosphorylation (*p* > 0.05) (Figure 5b–e).

Functional assays demonstrated that BIRB-796 markedly reduced ROS accumulation induced by *C. acnes* supernatant and restored SOD activity (*p* < 0.05) (Figure 5f–h). Additionally, BIRB-796 decreased apoptosis, downregulated cleaved caspase-3 and Bax expression, and upregulated Bcl-2 expression (*p* < 0.05) (Figure 5i–n). Moreover, BIRB-796 reversed the effects of *C. acnes* supernatant on ECM metabolism by increasing aggrecan and collagen II expression and decreasing MMP-13 and MM-P3 expression (*p* < 0.05) (Figure 5o–v). These results confirm that *C. acnes* supernatant induces oxidative stress, apoptosis, and ECM degradation in NP cells primarily through activation of the p38 MAPK signaling pathway.

### 2.4. Protective Effect of Wogonin on C. acnes Supernatant-Mediated Injury to NP Cells and IDD

#### 2.4.1. Wogonin Attenuates *C. acnes* Supernatant-Mediated Injury in NP Cells In Vitro

**Figure 6:**
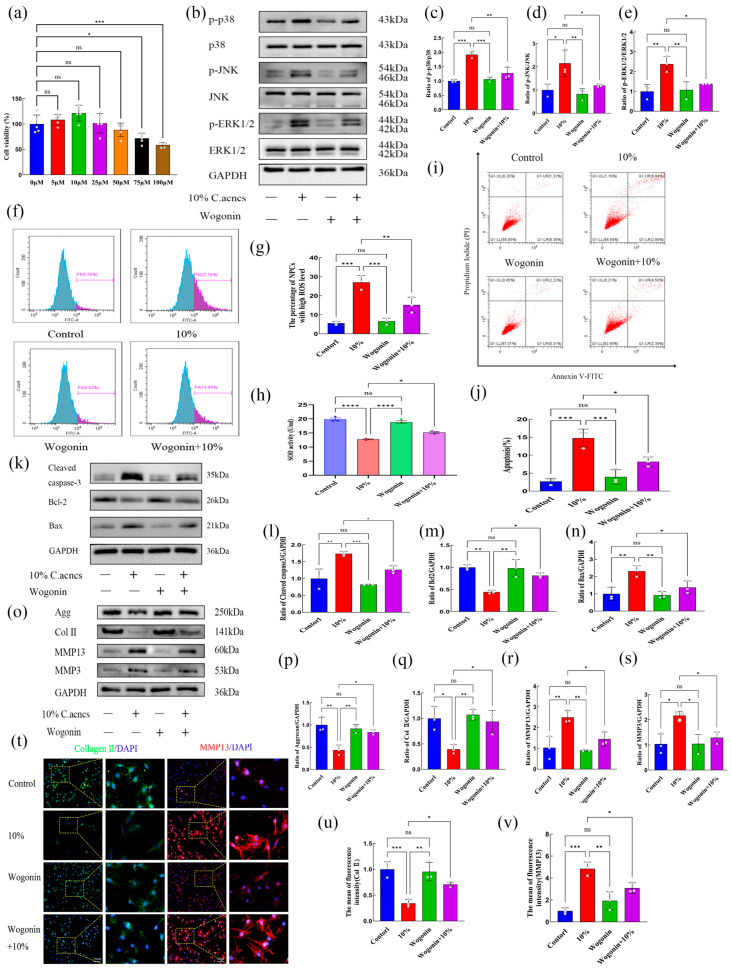
Effects of Wogonin on *C. acnes* supernatant-induced injury in rat NP cells in vitro. (**a**). Effect of Wogonin on NP cell viability. (**b**–**e**). Western blot analysis and quantification of MAPK signaling pathway proteins following Wogonin treatment. (**f**–**h**). ROS levels, quantitative analysis, and SOD activity after Wogonin treatment. (**i**–**n**). Flow cytometric analysis and quantification of apoptosis, and Western blot analysis of apoptosis-related proteins following Wogonin treatment. (**o**–**v**). Western blot analysis, quantitative analysis, and immunofluorescence detection of ECM-related markers after Wogonin treatment. ns: No statistical significance, * *p* < 0.05, ** *p* < 0.01, *** *p* < 0.001, **** *p* < 0.0001.

Cytotoxicity assays indicated that Wogonin has minimal impact on rat NP cell viability, with optimal survival observed at 10 μM (Figure 6a). This concentration was used in subsequent experiments. Western blot analysis demonstrated that Wogonin significantly inhibited the phosphorylation of p38 (p-p38), JNK (p-JNK), and ERK1/2 (p-ERK1/2) induced by *C. acnes* supernatant (*p* < 0.05) (Figure 6b–e), confirming its ability to suppress MAPK pathway activation.

Functional assays further showed that Wogonin markedly reduced ROS accumulation and restored SOD activity in NP cells exposed to *C. acnes* supernatant (*p* < 0.05) (Figure 6f–h). Wogonin also decreased apoptotic cell proportions, downregulated cleaved caspase-3 and Bax expression, and upregulated Bcl-2 expression (*p* < 0.05) (Figure 6i–n). Additionally, Wogonin mitigated ECM degradation by increasing aggrecan and collagen II expression while reducing MMP-13 and MMP-3 expression (*p* < 0.05) (Figure 6o–v). Collectively, these in vitro results indicate that Wogonin alleviates oxidative stress, apoptosis, and ECM degradation in NP cells induced by *C. acnes* supernatant.

#### 2.4.2. Wogonin Ameliorates *C. acnes*-Induced IDD In Vivo

**Figure 7:**
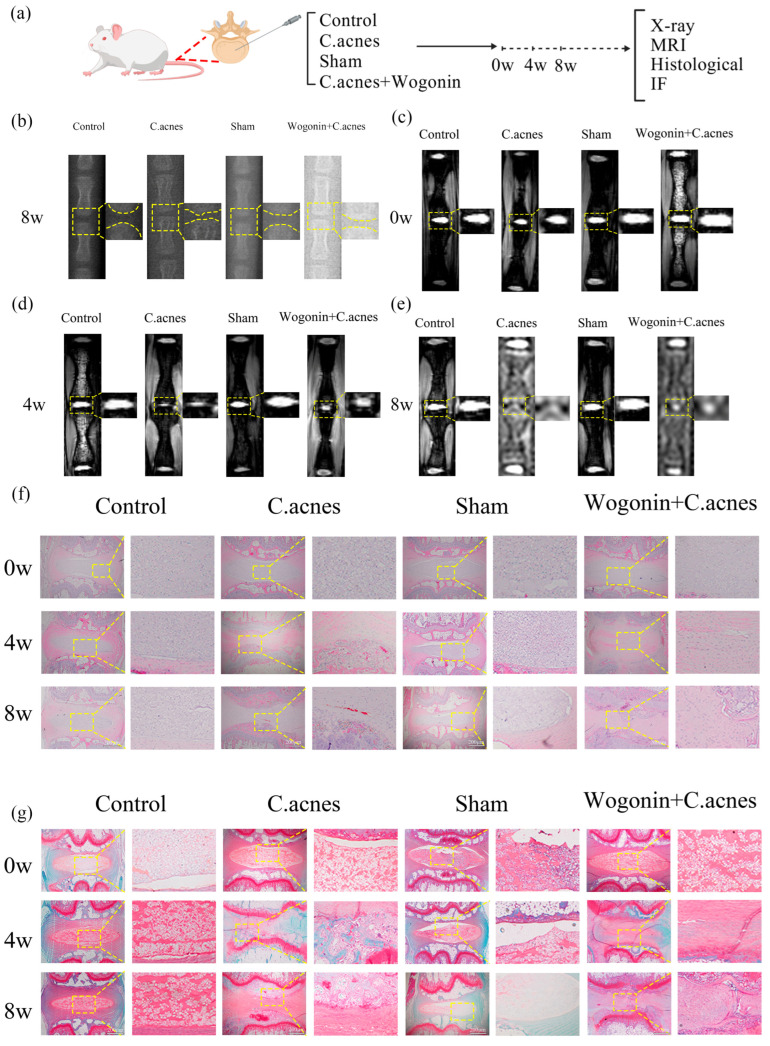
Imaging and histological evaluation of Wogonin-mediated repair of *C. acnes*-induced intervertebral disc degeneration (IDD) in rats. (**a**). Schematic diagram of the in vivo experimental model. (**b**). X-ray imaging results. (**c**–**e**). MRI findings. (**f**,**g**). Hematoxylin and eosin (H&E) and Safranin O–Fast Green staining.

Radiographic analyses at 8 weeks post-surgery revealed a significant reduction in intervertebral space height in the *C. acnes* group compared with control and sham groups. In the Wogonin + *C. acnes* group, intervertebral space height, although lower than control, was significantly higher than that of the *C. acnes* group (Figure 7b). MRI assessments showed that at 4 weeks post-surgery, T2-weighted NP signal intensity was markedly reduced in the *C. acnes* group, whereas the Wogonin + *C. acnes* group exhibited significantly higher signal intensity. By eight weeks, T2-weighted signals in the *C. acnes* group were further decreased, with structural disruption of the IVD. The Wogonin + *C. acnes* group maintained a higher signal than the *C. acnes* group, despite remaining lower than control (Figure 7c–e).

**Figure 8:**
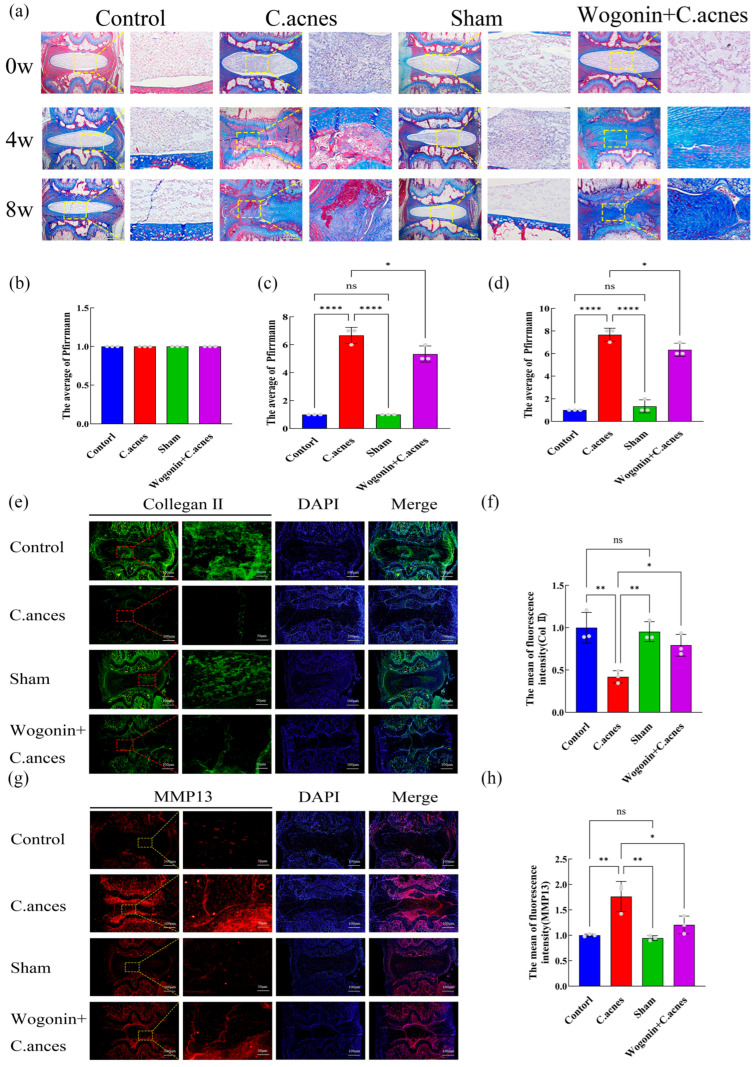
Histological staining and immunofluorescence analysis of Wogonin-mediated repair of *C. acnes*-induced IDD in rats. (**a**). Masson’s trichrome staining. (**b**–**d**). Modified Pfirrmann grading. (**e**–**h**). Immunofluorescence detection and quantitative analysis of ECM synthesis and degradation markers (collagen II and MMP-13) at 8 weeks post-surgery. ns: No statistical significance, * *p* < 0.05, ** *p* < 0.01, **** *p* < 0.0001.

Histological evaluation demonstrated that at 4 weeks, the *C. acnes* group displayed reduced intervertebral space height, diminished NP tissue, and inflammatory cell infiltration, all of which were markedly alleviated by Wogonin treatment. At 8 weeks, the *C. acnes* group showed pronounced NP tissue loss, endplate destruction, intervertebral space narrowing, and vascular invasion. In contrast, the Wogonin + *C. acnes* group exhibited substantially improved histopathology (Figure 7f,g and Figure 8a). Modified Pfirrmann grading corroborated these findings (*p* < 0.05) (Figure 8b–d). Immunofluorescence analysis revealed that collagen II expression was significantly decreased and MMP-13 expression increased in the *C. acnes* group, whereas Wogonin treatment reversed these alterations (*p* < 0.05) (Figure 8e–h). Together, these in vivo results confirm that Wogonin effectively mitigates *C. acnes*-induced IDD.

## 3. Discussion

IDD is the primary pathological basis of LBP. Its high prevalence and associated disability impose substantial burdens on global public health and healthcare systems. Traditional research has focused on classical mechanisms, including mechanical stress, oxidative stress, inflammatory responses, and cellular aging. However, these factors do not fully explain the etiology in certain IDD patients, particularly younger individuals, suggesting the involvement of previously unrecognized pathogenic pathways [1,31]. Recently, the potential role of low-toxicity anaerobic bacterial infections in IDD has garnered increasing attention. Among these, *C. acnes* has emerged as a candidate pathogen due to its frequent detection in degenerated IVD tissues [21]. In this study, we established a three-pronged experimental system—comprising clinical samples, in vitro cell experiments, and in vivo animal models—to investigate the pathological mechanisms by which *C. acnes* supernatant induces oxidative stress, apoptosis, and ECM degradation in NP cells through activation of the MAPK signaling pathway. Furthermore, we demonstrated that Wogonin can mitigate *C. acnes*-induced IDD progression by inhibiting this signaling pathway in both in vitro and in vivo models. This work advances the theoretical understanding of infection-associated IDD etiology and identifies potential therapeutic targets and candidate drugs for the precision treatment of *C. acnes*-related IDD, highlighting its academic novelty and translational potential.

### 3.1. Clinical Association and Pathological Significance of C. acnes in IDD

The role of infectious factors in IDD has received increasing attention. In this study, clinical samples from 73 patients with IDD were cultured, revealing that 13.7% of degenerated IVD tissues were positive for *C. acnes*, consistent with previously reported detection rates (15.5%) [21]. These findings further support the clinical association between low-toxicity anaerobic bacteria and IDD. Importantly, our study is the first to identify a significant positive correlation between *C. acnes* infection and both younger age as well as Modic changes, whereas no significant associations were observed with gender, comorbidities, degenerated segments, or Pfirrmann grading. This challenges the traditional view that IDD predominantly affects middle-aged and older adults and provides a new perspective on the pathogenesis of IDD in younger populations. Previous studies have suggested mechanisms underlying this association. Chen et al. reported that younger IDD patients have a higher incidence of annular tears, potentially facilitating *C. acnes* entry into the IVD [32]. Furthermore, Tang et al. found that IDD patients with *C. acnes*-positive IVD tissues were significantly younger than those with negative cultures, suggesting that the IVD microenvironment in younger patients favors bacterial survival and proliferation [33]. Our previous meta-analysis also demonstrated that *C. acnes*-positive patients were younger than those with negative cultures [34]. Collectively, these findings indicate that a relatively preserved vascular supply and a higher incidence of annular tears in younger patients create anatomical and microenvironmental conditions conducive to *C. acnes* colonization, suggesting that infection may be a key initiating factor in young IDD patients.

Modic changes—MRI signal abnormalities of the vertebral endplates and subendplate bone—are closely associated with inflammatory responses in IDD and bone metabolic disorders [17]. Aghazadeh et al. reported that 78.3% of patients with *C. acnes*-positive IVD tissues exhibited concomitant Modic changes, suggesting bacterial dissemination from the IVD to adjacent vertebral bodies may contribute to these changes [35]. Conversely, Wedderkopp et al. reported no significant association between *C. acnes* and Modic type I changes [36]. Albert et al., however, established a causal relationship in a cohort study, indicating that *C. acnes* infection increases the likelihood of adjacent vertebral Modic type I changes [37]. Current evidence increasingly supports a link between *C. acnes* infection and Modic changes, potentially mediated by oxidative stress and inflammatory responses in NP cells that affect bone metabolism at the endplates. Clinically, this association provides important reference indicators for identifying high-risk populations through imaging and introduces an infectious dimension to the etiological evaluation of IDD. Therefore, young LBP patients with Modic changes should be considered at elevated risk for *C. acnes* infection, providing critical guidance for targeted clinical assessment and intervention.

### 3.2. Molecular Mechanisms of NP Cell Damage Mediated by C. acnes Supernatant

Since *C. acnes* was proposed as a contributing factor to IDD, numerous studies have investigated whether its presence in IVD tissue reflects true infection and the in vivo pathogenic mechanisms of *C. acnes*. These studies have also identified distinct molecular pathways through which *C. acnes* may promote IDD. Tang et al. demonstrated that co-culturing *C. acnes* with NP cells increases ROS levels, NLRP3 expression, and pyroptosis markers. He et al. further showed that *C. acnes* induces NP cell pyroptosis via an NLRP3-dependent pathway, contributing to IDD. Lin et al. confirmed that *C. acnes* infection activates iNOS/NO and COX-2/PGE2 through a ROS-dependent NF-κB pathway in IVD tissues, promoting disc degeneration. Zhang et al. reported that *C. acnes* enhances MMP-1 expression and suppresses TIMP-1 expression via NF-κB signaling, further contributing to ECM disruption [38,39,40,41]. Collectively, these findings indicate that the molecular mechanisms by which *C. acnes* drives IDD are complex, incompletely understood, and likely involve multiple interacting pathways.

Oxidative stress imbalance is a central pathological feature of IDD, wherein excessive ROS accumulation coupled with impaired antioxidant defenses leads to functional damage in NP cells [42]. In this study, *C. acnes* supernatant was identified as a key pathogenic factor that dose-dependently damages NP cells. Supernatant concentrations of 10% or higher significantly elevated intracellular ROS levels and inhibited SOD activity, disrupting cellular redox homeostasis. Additionally, pro-apoptotic molecules, including cleaved caspase-3 and Bax, were upregulated, while the anti-apoptotic molecule Bcl-2 was downregulated, inducing apoptosis in NP cells. *C. acnes* supernatant also suppressed the expression of ECM synthesis-related molecules, such as aggrecan and type II collagen, while increasing ECM-degrading enzymes, including MMP-3 and MMP-13, leading to a disrupted ECM metabolic balance. These findings collectively demonstrate that *C. acnes* supernatant promotes IDD through a cascade involving oxidative stress, apoptosis, and ECM degradation, highlighting multiple potential targets for therapeutic intervention.

The MAPK signaling pathway has recently garnered attention for its role in transducing extracellular stimuli into cellular responses, including proliferation, migration, differentiation, and survival [43]. ROS can activate the MAPK pathway by oxidizing cysteine residues of key enzymes, inducing structural changes that trigger downstream signaling. Notably, the Thr-Gly-Tyr dual phosphorylation site of p38 MAPK is highly sensitive to oxidative modification, and ROS promote its phosphorylation to initiate signaling [44,45,46]. The MAPK pathway is closely linked to both apoptosis and ECM degradation, serving as a critical regulator of these processes [47,48,49]. Among the three major sub-pathways of the MAPK family, p38 MAPK is the most critical and effective signaling pathway mediating oxidative stress damage, playing a dominant regulatory role in the accumulation of ROS, redox imbalance, mitochondrial dysfunction, and the degeneration and apoptosis of nucleus pulposus cells. Oxidative stress can preferentially activate the p38 pathway through the upstream ASK1 kinase, further inhibiting the Nrf2 antioxidant signaling axis and amplifying the vicious cycle of oxidative damage [50,51]. To elucidate the molecular mechanisms underlying NP cell damage induced by *C. acnes* supernatant, RNA sequencing analysis revealed the MAPK pathway as a core upstream regulator of this pathogenic cascade. *C. acnes* supernatant markedly increased phosphorylation of p38, JNK, and ERK1/2, with p38 MAPK identified as the primary functional branch. The p38-specific inhibitor BIRB-796 effectively reversed *C. acnes* supernatant-induced oxidative stress, apoptosis, and ECM degradation, whereas inhibition of JNK and ERK1/2 showed no significant effect. These results refine the molecular network of *C. acnes*-mediated IDD and identify key signaling targets for potential therapeutic development, addressing current gaps in understanding the pathogenic mechanisms of *C. acnes*.

### 3.3. Clinical Value of Wogonin in the Treatment of C. acnes-Associated IDD

Antibiotics were previously considered a potential treatment for IDD associated with bacterial infection. However, the global resistance rate of *C. acnes* has exceeded 50%. Moreover, the avascular structure of IVDs limits antibiotic penetration into affected tissues, preventing the achievement of effective antibacterial concentrations. Prolonged antibiotic use can also cause significant complications, including disruption of gut microbiota and the emergence of antibiotic-resistant bacteria. Consequently, a “de-antibiotic strategy” has become an important focus of research in this field [52,53,54]. Oxidative stress is a fundamental pathological process involved in the onset and progression of IDD. It accelerates IDD development through various mechanisms, including DNA damage in nucleus pulposus cells, disruption of mitochondrial function, and modulation of inflammatory factor release [8,55]. Research focused on the prevention and treatment of IDD via reactive oxygen species (ROS) scavenging and antioxidant strategies has long been a topic of considerable interest. Melatonin, a mitochondria-targeted antioxidant, enhances the activity of superoxide dismutase and glutathione, thereby protecting NP cells from oxidative stress-induced mitochondrial dysfunction and apoptotic damage. Furthermore, melatonin improves the imbalance in type II collagen synthesis induced by hydrogen peroxide [56,57]. In addition to its well-known hypoglycemic effects, metformin can downregulate the p53/p21/Rb and p16/Rb signaling axes, thereby inhibiting apoptosis and matrix degradation in NP cells while also alleviating oxidative stress damage by promoting autophagy [58]. Reduced glutathione (GSH), a tripeptide composed of glutamate, cysteine, and glycine, is the most abundant low-molecular-weight thiol compound and serves as a key endogenous antioxidant. Its prodrug, N-acetylcysteine (NAC), effectively reduces intracellular ROS levels and inhibits the activation of the MAPK and mTOR pathways in both human and rat nucleus pulposus cells. Additionally, NAC blocks hydrogen peroxide-induced apoptosis and calcification of the cartilage endplate by downregulating the ROS/MAPK/NF-κB signaling pathway [59,60]. Natural products are increasingly recognized as promising therapeutic candidates for IDD because of their low toxicity, favorable biocompatibility, and multi-target regulatory properties [61,62]. Resveratrol, a non-flavonoid polyphenolic compound, is a phytoalexin produced by plants in response to stress. It can reduce levels of inflammatory factors such as IL-1β, IL-6, IL-8, and TNF-α by inhibiting the JAK/STAT3 pathway and the ROS-activated NF-κB signaling pathway, thereby alleviating inflammation in the intervertebral discs [63]. Naringin, a dihydroflavonoid compound, possesses significant anti-inflammatory and antioxidant activities. It can inhibit apoptosis in annulus fibrosus cells and reduce the release of inflammatory mediators by blocking the ROS/NF-κB pathway, thereby delaying intervertebral disc degeneration [64,65]. In addition, quercetin, a well-established flavonoid antioxidant, can scavenge ROS and inhibit inflammation by activating the Nrf2/NF-κB signaling axis. This action improves damage to nucleus pulposus cells and slows the progression of IDD [66].

Wogonin, a natural flavonoid isolated from *Scutellaria baicalensis*, exhibits a broad range of pharmacological activities, including antioxidant, anti-inflammatory, and anti-apoptotic effects. It has demonstrated therapeutic potential in several diseases, such as diabetic nephropathy, inflammatory bowel disease, and osteoarthritis [67,68,69]. However, its role in IDD associated with *C. acnes* infection has not previously been investigated. In the present study, we demonstrate for the first time that Wogonin shows no significant cytotoxicity toward NP cells at a concentration of 10 μM. Wogonin inhibits pathological signaling induced by *C. acnes* supernatant through upstream suppression of p38, JNK, and ERK1/2 phosphorylation within the MAPK signaling pathway. This inhibition reduces intracellular ROS levels, restores SOD activity, and alleviates oxidative stress. Furthermore, Wogonin downregulates pro-apoptotic molecules while upregulating anti-apoptotic factors, thereby suppressing apoptosis in NP cells and restoring the balance between ECM synthesis and degradation. In vivo experiments further demonstrate that Wogonin reduces *C. acnes* persistence in rat tail vertebrae and alleviates pathological changes associated with disc degeneration, including reduced disc height and loss of NP tissue. Wogonin also improves abnormal MRI T2-weighted signals and reduces histological inflammatory infiltration. In addition, it upregulates type II collagen expression while downregulating MMP-13 expression, thereby restoring ECM metabolic balance. The intervertebral discs in adults are typically avascular tissues, and the low bioavailability of flavonoid compounds presents a significant challenge for the clinical translation of Wogonin [70]. Recent studies indicate that employing hydrogels responsive to the pathological microenvironment for drug delivery can facilitate the slow and controlled release of drugs under the pathological conditions associated with IDD, such as local acidity and elevated levels of ROS. This approach not only extends the duration of drug action but also enhances bioavailability, mitigates systemic adverse effects, and simplifies the administration process, making it more suitable for clinical applications [71]. Furthermore, integrating nanodelivery systems to formulate Wogonin into nanoparticles or nanomicelles can significantly improve the drug’s targeting and tissue penetration, thereby increasing its accumulation efficiency in nucleus pulposus cells and further optimizing therapeutic effects [72]. Consequently, the construction of an efficient and minimally invasive delivery system for Wogonin will be a key focus of our future research efforts. Collectively, these findings establish, for the first time, a link between Wogonin and the treatment of IDD related to *C. acnes* infection highlighting its potential as a natural alternative to antibiotics and providing a new therapeutic direction for addressing antibiotic resistance in *C. acnes*-associated spinal disorders.

This study has several limitations. First, it examined only the pathogenic effects of *C. acnes* supernatant and did not isolate or verify the specific pathogenic components within the supernatant, such as lipopolysaccharides or exotoxins. Future studies should employ techniques such as mass spectrometry to identify the key pathogenic molecules. Second, the animal model used in this study was a rat tail IVD injection model, which differs from the anatomical structure and mechanical environment of human lumbar IVDs. Our research group is currently developing a degeneration model that more closely resembles human pathology. We look forward to the development of animal models that more closely mimic the pathological and physiological mechanisms of IDD in humans in the future. Third, the clinical sample size in this study was relatively small. Future research should include larger multicenter cohorts to further validate the association between *C. acnes* infection and the clinical features of IDD.

## 4. Materials and Methods

### 4.1. Collection of Human NP Tissue Specimens and Bacterial Culture

Human IVD tissue specimens were obtained from inpatients undergoing surgery in the Department of Spine Surgery at Lanzhou University Second Hospital. Specimens were collected and classified according to the Pfirrmann grading system [73]. Exclusion criteria included a documented history of infection or antibiotic use within 1 month prior to surgery, previous vertebral or IVD infections, tumor-related cases, and failed spinal surgeries. To minimize the risk of contamination, tissue specimens were collected intraoperatively under sterile conditions. Following removal of the IVD and a small amount of adjacent muscle tissue, specimens were immediately transferred into Eppendorf tubes containing brain–heart infusion broth for both aerobic and anaerobic culture. The turbidity of the culture medium was monitored continuously. When turbidity was observed, a small volume of the culture medium was inoculated onto Columbia blood agar plates (Beckman Biotechnology Co., Ltd., Changde, China.) and incubated under anaerobic conditions until distinct bacterial colonies formed. Bacterial strains were identified using matrix-assisted laser desorption/ionization time-of-flight mass spectrometry (MALDI-TOF MS). If no turbidity was observed after 14 consecutive days of incubation, the culture was terminated.

### 4.2. Gram Staining

Colonies in the active growth phase isolated from Columbia blood agar plates were used to prepare bacterial suspensions. The suspensions were evenly spread onto clean glass slides, air-dried, and heat-fixed. Gram staining was performed using a Gram staining kit (Biosharp, Beijing, China) according to the manufacturer’s instructions. After staining, the slides were examined under oil immersion microscopy (Olympus, Tokyo, Japan). Gram-positive bacteria appeared blue-violet, whereas Gram-negative bacteria appeared red.

### 4.3. Experimental Animals and Primary Extraction of NP Cells

Healthy male Sprague–Dawley rats aged 4–8 weeks (200–220 g) were obtained from the Lanzhou Veterinary Research Institute of the Chinese Academy of Agricultural Sciences (animal license number: SCXK [Gansu] 2020-0002). Primary NP cells were isolated from rat tail vertebrae using aseptic techniques. The procedure included the following steps: (1) sterilization of experimental instruments; (2) isolation and extraction of NP tissue; (3) enzymatic digestion of NP tissue with type II collagenase (Proteintech, Wuhan, China); and (4) culture of isolated cells in DMEM/F-12 complete medium supplemented with 11% fetal bovine serum (FBS), with continuous monitoring of cell adhesion and periodic medium replacement. When the NP cell density in the culture flasks exceeded 90%, the cells were passaged for further experiments.

### 4.4. Preparation of Experimental Strains, C. acnes-Conditioned Medium, and Co-Culture with NP Cells

The *C. acnes* strain used in this study was obtained from the Guangdong Microbial Culture Collection Center. The cultured bacterial suspension was diluted 10^4^-fold, and the bacterial concentration was adjusted to a final density of 4 × 10^7^ CFU/mL based on absorbance measurements at 600 nm. The bacterial suspension was subsequently centrifuged at 10,000 rpm for 15 min to separate the cells from the supernatant. The supernatant was filtered through a 0.22 μm membrane filter and stored at 4 °C for later use as *C. acnes*-conditioned medium. NP cells were prepared as a cell suspension at a concentration of 1–3 × 10^4^ cells/mL and incubated in a 37 °C cell culture incubator for 24 h. When the cell confluence reached approximately 80%, different concentrations of *C. acnes*-conditioned medium were added, and the cells were co-cultured for an additional 24 h.

### 4.5. Cell Counting Kit-8 (CCK-8) Assay

NP cells were prepared as a suspension and seeded into 96-well plates at a density of 1 × 10^4^ cells per well in 100 μL of medium. The plates were incubated at 37 °C for 12 h to allow cell adhesion. Subsequently, different concentrations of *C. acnes* supernatant (2.5%, 5%, 7.5%, 10%, 15%, 20%, 25%, and 30%) were added, and the cells were co-cultured for 24 h. After incubation, 10 μL of CCK-8 solution (Beyotime, Shanghai, China) was added to each well. The plates were then incubated at 37 °C in the dark for 1–4 h, during which color changes were monitored (a deeper orange-yellow color indicates higher cell viability). Finally, the optical density (OD) at 450 nm was measured using a microplate reader (BioTek Instruments, Winooski, VT, USA).

### 4.6. Live/Dead Cell Staining

NP cells were digested and resuspended using standard methods and seeded at a density of 1.0 × 10^5^ cells per well in six-well plates. The plates were incubated at 37 °C for 12 h to allow cell adherence. Cells were then treated with different concentrations of *C. acnes* supernatant and incubated at 37 °C for 24 h. After treatment, the culture medium was discarded and replaced with fresh medium containing a Calcein-AM/PI staining solution for live and dead cells (Beyotime, Shanghai, China). The cells were incubated at room temperature in the dark for 30 min. Fluorescence microscopy was used to observe the cells, with live cells exhibiting green fluorescence and dead cells exhibiting red fluorescence.

### 4.7. Analysis of Oxidative Stress and Apoptosis by Flow Cytometry

(1) Detection of Oxidative Stress in NP Cells

Oxidative stress levels in the cells were assessed using a ROS detection kit (Beyotime, Shanghai, China). NP cells were seeded in six-well plates at a density of 1.0 × 10^5^ cells per well and treated with different concentrations of *C. acnes* supernatant for 24 h at 37 °C. After treatment, cells were collected into flow cytometry tubes and incubated with the ROS detection reagent at room temperature for 30 min in the dark. Fluorescence intensity was analyzed using a Beckman CytoFLEX flow cytometer (Beckman Coulter Life Sciences, Brea, CA, USA), and the data were processed using FlowJo 10 software (Tree Star, Ashland, OR, USA).

(2) Detection of Apoptosis in NP Cells

The cell seeding and treatment procedures were the same as described above. Cells were digested with 0.25% trypsin without EDTA (Gibco, Grand Island, NY, USA) and centrifuged at 1200 rpm for 5 min to collect the cell pellet. Apoptosis was detected using an apoptosis detection kit (Leagene, Beijing, China) according to the manufacturer’s instructions. Samples were analyzed using a Beckman CytoFLEX flow cytometer, and the data were processed using FlowJo 10 software.

### 4.8. Detection of Superoxide Dismutase (SOD) Activity

Superoxide dismutase (SOD) activity in NP cells was measured using an SOD detection kit (Beyotime, Shanghai, China). The cell seeding and treatment procedures were consistent with those described above.

After treatment, cell supernatants were collected and centrifuged at 12,000 rpm for 5 min. The resulting supernatant was transferred to a 96-well plate, and SOD detection reagents were added according to the manufacturer’s protocol. The mixture was incubated in a 37 °C water bath for 30 min. OD values were measured at 450 nm using a microplate reader, and the data were recorded for subsequent analysis.

### 4.9. Western Blot

Total protein from NP cells was extracted using RIPA lysis buffer containing 1% phenylmethanesulfonyl fluoride and 1% phosphatase inhibitor (Solarbio). Protein concentrations were determined using a BCA protein assay kit (Beyotime). Equal amounts of protein were separated by SDS-PAGE and transferred onto polyvinylidene fluoride membranes. The membranes were blocked with blocking buffer (Beyotime) and incubated overnight at 4 °C with primary antibodies. After washing, the membranes were incubated with HRP-conjugated secondary antibodies for 2 h at room temperature.

Following Tris-buffered saline with Tween-20 washes, protein bands were visualized using an enhanced chemiluminescent reagent (Bio-Rad Laboratories, Hercules, CA, USA) and captured using a gel imaging system (Bio-Rad, USA). Band intensities were quantified using ImageJ software (Version 1.53t, National Institutes of Health, Bethesda, MD, USA). Details of the antibodies used are listed in Supplementary Table S1.

### 4.10. Immunofluorescence Staining of Cells and Tissue

(1) Cell Immunofluorescence

NP cells were fixed with 4% paraformaldehyde (Beyotime, Shanghai, China) for 15 min, washed with phosphate-buffered saline, and permeabilized with Triton X-100 (Servicebio, Wuhan, China) for 25 min. The cells were then blocked with 5% goat serum for 1 h at room temperature and incubated with the primary antibody overnight at 4 °C. After washing, fluorescein isothiocyanate- or Cy3-conjugated secondary antibodies (1:200 dilution) were added and incubated at 37 °C for 60 min. The samples were mounted with antifade mounting medium containing DAPI (Solarbio, Beijing, China), and images were captured using a fluorescence microscope (Olympus, Tokyo, Japan).

(2) Tissue Immunofluorescence

Rat IVD tissue sections were immersed in 1× antigen retrieval solution and heated in a microwave oven until boiling, followed by continued heating for 10 min. The tissue area was outlined using an immunohistochemistry pen, and the sections were blocked with 5% goat serum for 30 min at room temperature. The sections were then incubated with primary antibodies overnight at 4 °C, followed by incubation with species-specific fluorescent secondary antibodies for 1 h at room temperature. Finally, the sections were mounted with antifade mounting medium containing DAPI, covered with coverslips, and observed under a fluorescence microscope for image acquisition.

### 4.11. RNA Sequencing and Analysis

NP cells were co-cultured with 10% *C. acnes* supernatant for 24 h, with three biological replicates per group. Total RNA was extracted from both the infected and control NP cells. RNA sequencing and subsequent data analysis were performed by Wuhan Feisha Gene Company.

### 4.12. Animal Models and Imaging Assessments

Healthy male Sprague–Dawley rats were randomly assigned to four groups: control, *C. acnes* supernatant, sham, and Wogonin + *C. acnes* supernatant. Sterile Wogonin (20 mg; MeilunBio, Dalian, China) was dissolved in 704 μL dimethyl sulfoxide (DMSO) to produce a 100 mM stock solution, which appeared as a yellow transparent solution and was stored at −80 °C in the dark. Before use, the stock solution was thawed at room temperature and diluted with DMSO to a working concentration of 50 μM. Following standard anesthesia procedures, the IVD space between the seventh and eighth tail vertebrae was identified. Interventions were performed using a 31-gauge microsyringe. The sham group received a disc puncture without fluid injection. The *C. acnes* group received 5 μL of *C. acnes* supernatant and 2 μL of physiological saline, while the Wogonin + *C. acnes* group received 5 μL of *C. acnes* supernatant together with 2 μL of 50 μM Wogonin solution [74]. MRI examinations were performed with rats in the prone position at postoperative weeks 0, 4, and 8 using a uMR 9.4T MRI system (United Imaging Life Science Instrument, Wuhan, China) to obtain T2-weighted images. In addition, X-ray examinations were conducted at postoperative week 8 using a SONIALVISION R300 multifunctional dynamic flat-panel X-ray system (Shimadzu Corporation, Kyoto, Japan).

### 4.13. Histological Assessment

Rats were euthanized by an overdose injection of pentobarbital at postoperative weeks 0, 4, and 8, and IVD specimens were collected. The specimens were fixed in 4% paraformaldehyde for 1 week, decalcified in EDTA decalcification solution (Servicebio, Wuhan, China) for 4 weeks, and embedded in paraffin. Sagittal sections (5 μm) were prepared for histological analysis. Sections were stained with hematoxylin and eosin (H&E), Safranin O/Fast Green, and Masson’s trichrome (Solarbio, Beijing, China). All staining procedures were performed according to the manufacturer’s instructions, and images were captured using a light microscope (Olympus, Tokyo, Japan).

### 4.14. Ethical Approval

All animal experiments were conducted in accordance with the ARRIVE guidelines and were approved on 17 December 2025 by the Animal Care and Use Committee of the Second Hospital of Lanzhou University (Approval No. D2025-896). Human sample collection and related experiments complied with the Declaration of Helsinki and were approved by the Ethics Review Committee of the Second Hospital of Lanzhou University (Approval No. 2025A-341). Written informed consent was obtained from all participants.

### 4.15. Statistical Analysis

All experiments were independently repeated at least three times. Data were normally distributed with homogeneity of variance and were presented as mean ± standard deviation; some tissue specimen data were reported as mean ± standard error (SE). Statistical graphs were generated using GraphPad Prism 10 software. Comparisons between two groups were performed using independent-samples *t*-tests, while one-way analysis of variance was used for comparisons among three or more groups. *p*-values represented by *, **, and *** correspond to *p* < 0.05, *p* < 0.01, and *p* < 0.001, respectively. A *p* value < 0.05 was considered statistically significant.

## 5. Conclusions

**Figure 9:**
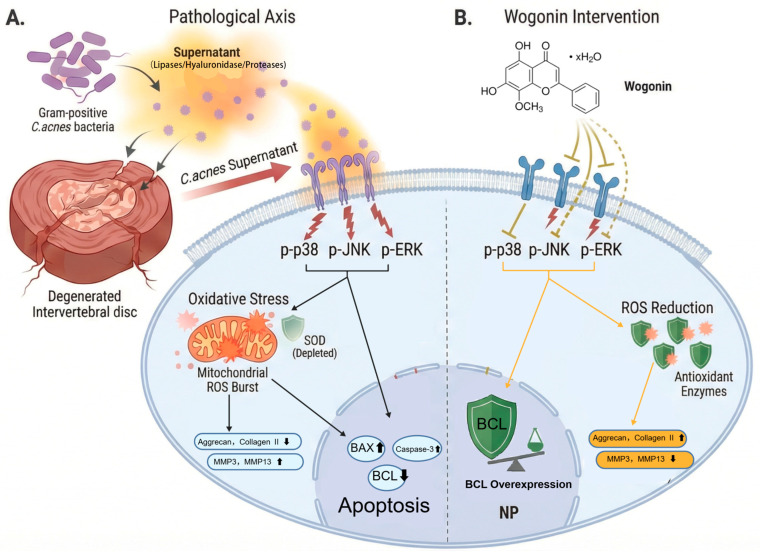
Proposed mechanism by which Wogonin alleviates *C. acnes* supernatant-induced intervertebral disc degeneration through regulation of the MAPK signaling pathway. (**A**) The supernatant of *C. acnes* induces oxidative stress in nucleus pulposus cells. (**B**) Wogonin can effectively reverse this process.

In summary, this study is the first to establish a clinical association between *C. acnes* infection, young age in patients with IDD, and Modic changes. It elucidates the molecular mechanism by which *C. acnes* supernatant induces oxidative stress, apoptosis, and ECM degradation in NP cells through activation of the p38 MAPK signaling pathway. Furthermore, both in vitro and in vivo results demonstrate that Wogonin can effectively attenuate *C. acnes*-mediated IDD by inhibiting MAPK signaling. The novelty of this study lies in linking low-virulence anaerobic bacterial infection to the pathogenesis of IDD and identifying the central regulatory role of the MAPK signaling pathway. In addition, this work identifies the therapeutic potential of Wogonin as a targeted intervention, providing new insights into the etiology and molecular mechanisms of IDD and offering a theoretical and experimental basis for the precise diagnosis and treatment of IDD associated with *C. acnes* infection in clinical practice (Figure 9).

## Figures and Tables

**Table 1:** Correlation between *C. acnes* positivity and patient characteristics.

Factors	Positive (*n* = 10)	Negative (*n* = 63)	X^2^/t	*p*
Gender			1.983	>0.05
Male	7 (70.00%)	29 (46.03%)		
Female	3 (30.00%)	34 (53.97%)		
Age (years, x ± s)	45.90 ± 10.51	55.21 ± 13.35	2.099	<0.05
Medical history				
Diabetes	1 (10.00%)	9 (14.29%)	0.134	>0.05
Hypertension	2 (20.00%)	13 (20.63%)	0.002	>0.05
Degenerated segments			0.765	>0.05
L3—4	1 (10.00%)	6 (9.52%)		
L4—5	5 (50.00%)	40 (63.49%)		
L5—S1	4 (40.00%)	17 (26.98%)		
Pfirrmann grade			2.851	>0.05
III	6 (60.00%)	21 (33.33%)		
IV	3 (30.00%)	36 (54.14%)		
V	1 (10.00%)	6 (9.52%)		
Modic changes	6 (60.00%)	15 (23.81%)	3.891	<0.05

## Data Availability

The datasets used and/or analysed during the current study available from the corresponding author on reasonable request.

## Supplementary Materials

The following supporting information can be downloaded at: https://www.mdpi.com/article/10.3390/ijms27104249/s1.
