# Supplementary material for: Wogonin Ameliorates the Oxidative Stress, Apoptosis, and Extracellular Matrix Degradation of Nucleus Pulposus Cells Mediated by Cutibacterium acnes via the MAPK Signaling Pathway: An In Vivo and In Vitro Study
Source: Int J Mol Sci. 2026 May 10;27(10):4249. doi: 10.3390/ijms27104249 (PMC13206970; doi:10.3390/ijms27104249)
Supplement: Supplementary file 1 [file ijms-27-04249-s001.zip › ijms-4241964-supplementary.pdf]

Supplementary Table S1. The list of primary antibodies.

Name	Company
Anti-Cleaved-caspase3	Proteintech
Anti-Bcl-2	Proteintech
Anti-Bax	Proteintech
Anti-Aggreecan	Proteintech
Anti-Collagen II	Proteintech
Anti-MMP3	Proteintech
Anti-MMP13	Proteintech
Anti-p-p38	Cell Signaling Technology
Anti-p38	Cell Signaling Technology
Anti-p-JNK	Cell Signaling Technology
Anti-JNK	Cell Signaling Technology
Anti-p-ERK1/2	Proteintech
Anti-ERK1/2	Proteintech
GAPDH Monoclonal antibody	Proteintech
